# Defining Early Human NK Cell Developmental Stages in Primary and Secondary Lymphoid Tissues

**DOI:** 10.1371/journal.pone.0030930

**Published:** 2012-02-03

**Authors:** Diana N. Eissens, Jan Spanholtz, Arnold van der Meer, Bram van Cranenbroek, Harry Dolstra, Jaap Kwekkeboom, Frank W. M. B. Preijers, Irma Joosten

**Affiliations:** 1 Department of Laboratory Medicine - Laboratory of Medical Immunology, Radboud University Nijmegen Medical Centre, Nijmegen, The Netherlands; 2 Department of Laboratory Medicine - Laboratory of Hematology, Radboud University Nijmegen Medical Centre, Nijmegen, The Netherlands; 3 Laboratory of Gastroenterology and Hepatology, Erasmus Medical Centre, Rotterdam, The Netherlands; Centre de Recherche Public de la Santé (CRP-Santé), Luxembourg

## Abstract

A better understanding of human NK cell development *in vivo* is crucial to exploit NK cells for immunotherapy. Here, we identified seven distinctive NK cell developmental stages in bone marrow of single donors using 10-color flow cytometry and found that NK cell development is accompanied by early expression of stimulatory co-receptor CD244 *in vivo*. Further analysis of cord blood (CB), peripheral blood (PB), inguinal lymph node (inLN), liver lymph node (liLN) and spleen (SPL) samples showed diverse distributions of the NK cell developmental stages. In addition, distinctive expression profiles of early development marker CD33 and C-type lectin receptor NKG2A between the tissues, suggest that differential NK cell differentiation may take place at different anatomical locations. Differential expression of NKG2A and stimulatory receptors (e.g. NCR, NKG2D) within the different subsets of committed NK cells demonstrated the heterogeneity of the CD56^bright^CD16^+/−^ and CD56^dim^CD16^+^ subsets within the different compartments and suggests that microenvironment may play a role in differential *in situ* development of the NK cell receptor repertoire of committed NK cells. Overall, differential *in situ* NK cell development and trafficking towards multiple tissues may give rise to a broad spectrum of mature NK cell subsets found within the human body.

## Introduction

Natural killer (NK) cells are large CD56^+^CD3^−^ granular lymphocytes and are considered part of the innate immune system. NK cells can kill infected or malignant transformed cells without prior sensitization, and through the production of cytokines, such as IFN-γ, they form a bridge between innate and adaptive immune responses [1], [2]. NK cell reactivity is tightly regulated through a balance of signals between stimulatory and inhibitory receptors, a feature that is being exploited today for NK cell-based immunotherapy against cancer [3]. For this, a thorough understanding of human NK cell development *in vivo* is crucial.

Bone marrow (BM) is generally considered as the primary site for human NK cell development [4]–[7]. However, a complete pathway for NK cell development and maturation in BM has not been described and it may be possible that precursor NK cells traffic from BM to other tissues for terminal differentiation *in situ* [8]. In 2005, Freud *et al.* [9] identified a BM-derived CD34^+^ hematopoietic precursor cell residing in lymph nodes (LN) where further differentiation into CD56^bright^ cells could take place. Subsequently, they identified four discrete stages for human NK cell development within secondary lymphoid tissues (SLT) based on cell surface expression of CD34, CD117 and CD94: stage 1, CD34^+^CD117^−^CD94^−^; stage 2, CD34^+^CD117^+^CD94^−^; stage 3, CD34^−^CD117^+^CD94^−^; and stage 4, CD34^−^CD117^+/−^CD94^+^ [10]. Following NK cell development, commitment to the NK cell lineage takes place at stage 3, in which CD56 appears on the cell surface and gives rise to CD56^bright^ NK cells in stage 4. These data confirmed previous research describing the abundant presence of CD56^bright^ NK cells in SLT [11], [12]. In addition, we and others have shown that CD56^bright^ cells are the first mature NK cells to arise after allogeneic stem cell transplantation (SCT) [13], [14]. Overall, these data support a model of *in vivo* human NK cell development in which CD34^+^ NK cell precursors may traffic from BM to SLT where further differentiation into CD56^bright^ NK cells occurs. However, how these NK cell developmental stages correlate with NK cell subsets in other compartments of the human body (e.g. peripheral blood (PB), spleen (SPL)) remains unclear.

In this study, we identified seven distinctive NK cell developmental stages in bone marrow using 10-color flow cytometry and found that NK cell development is accompanied by early expression of stimulatory co-receptor CD244 *in vivo*. Furthermore, distinctive expression profiles of early development marker CD33 and the C-type lectin receptor NKG2A between different tissues, suggest that differential *in situ* NK cell differentiation may take place at different anatomical sites. Thus, differential *in situ* NK cell development and potential trafficking towards multiple tissues may give rise to a broad spectrum of mature NK cell subsets found within the human body. The findings presented here may serve as a fundamental basis for ongoing and future NK cell development studies and the development of NK cell generation protocols used for clinical purposes.

## Results

To identify human NK cell developmental stages within the different tissues and to analyze the distribution of different NK cell subsets and their NK cell receptor repertoire, we designed three 10-color flow cytometry (FCM) panels (Table 1). As BM is considered the origin of NK cell development [4]–[7], we first analyzed BM for the presence of NK cell developmental stages.

**Table 1:** Panels used for flow cytometry.

	FITC	PE	ECD	PC5.5	PC7	APC	APC-A700	APC-A750	PB	PO
1	CD34	CD133	CD3	CD159a	CD117	CD33	CD244	CD56	CD94	CD45
	*581*	*AC133*	*UCHT1*	*Z199.1.10*	*104D2D1*	*D3HL60.251*	*C1.7.1*	*N901*	*HP-3B1*	*J33*
2	CD16	CD159c	CD3	-	CD158b	CD158e1	CD158a	CD56	CD159a	CD45
	*DJ130c*	*134522*	*UCHT1*	*-*	*GL183*	*Z27.3.7*	*EB6.B.3.1.1*	*N901*	*Z199.1.10*	*J33*
3	CD16	CD336	CD3	CD337	CD335	CD314	CD244	CD56	-	CD45
	*DJ130c*	*Z231*	*UCHT1*	*Z25*	*BAB281*	*ON72*	*C1.7.1*	*N901*	-	*J33*

Displayed are the combinations of conjugated monoclonal antibodies (mAb) against specific antigens within each panel. In addition, the clone for each specific mAb is shown. Each panel was used for flow cytometric (FCM) analysis of bone marrow, cord blood, peripheral blood, inguinal LN, liver LN, and spleen samples of human donors (all n = 5). Thawed MNC fractions of the human tissue samples were assessed on a NaviosTM 10-color flow cytometer and analyzed using Kaluza Software® 1.0 (Beckman coulter). Panel 1 was used to identify different NK cell developmental stages based on CD34, CD117, CD94 and CD56 expression profiles.^10^ Additionally, expression of early development markers CD133 and CD33, stimulatory co-receptor 2B4 (CD244), and C-type lectin NKG2A were analyzed to refine the definition of the different NK cell developmental stages. Panel 2 and 3 were used to analyze the NK cell receptor repertoire of CD45^+^CD56^bright^CD16^+/−^CD3^−^ and CD45^+^CD56^dim^CD16^+^CD3^−^ NK cells consisting of inhibitory and stimulatory receptors. Inhibitory receptors contain KIR (CD158a, CD158b, CD158e1) and NKG2A (CD159a). Stimulatory receptors contain NCR (CD335/336/337), NKG2C (CD159c), NKG2D (CD314), and 2B4 (CD244).

### Identification of seven NK cell developmental stages in BM

**Figure 1:**
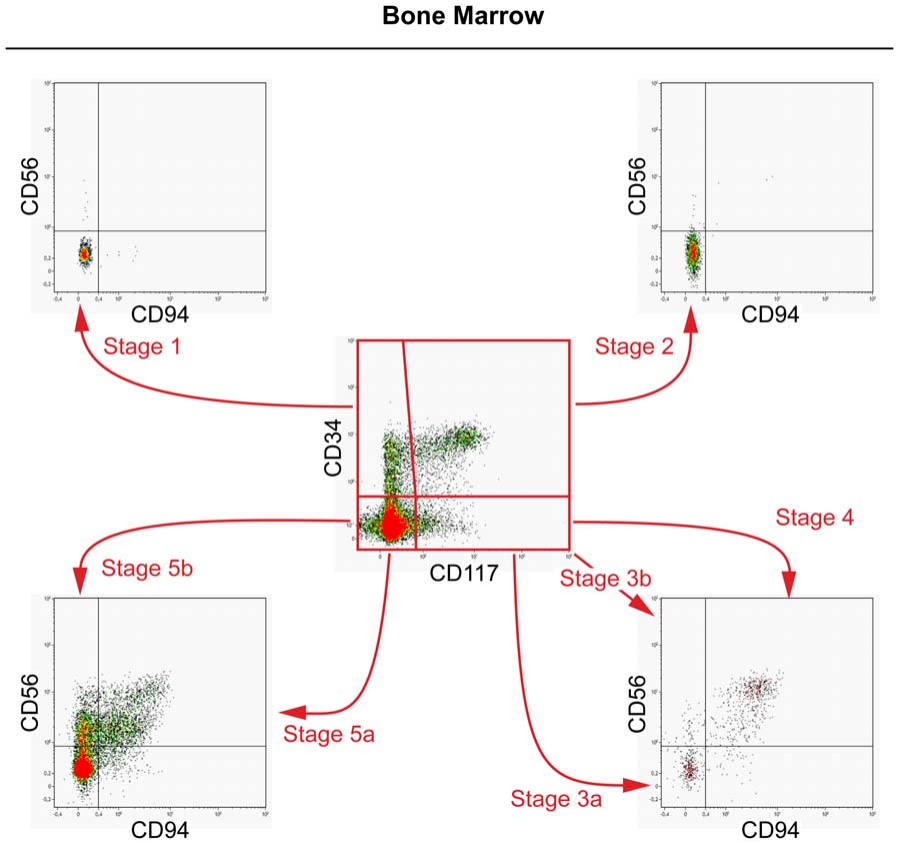
Identification of seven NK cell developmental stages in bone marrow (BM). Based on the stages defined in Table 1, we analyzed the presence of the different NK cell developmental stages in BM. Shown is one representative example (n = 5). Cells were gated on the CD45^+^CD3^−^ population within CD45^+^/SS gated cells to exclude T cells and endothelial cells from analysis. Subsequently, cell subsets were divided based on the expression of CD34 and CD117. From there, each subset was analyzed for CD56 and CD94 expression, leading to the identification of seven NK cell developmental stages: 1, 2, 3a, 3b, 4, 5a, 5b.

Distinct NK cell developmental stages can be characterized through expression analysis of CD34, CD117, CD94 and CD56 antigens [10]. Based on that, we gated our samples on the CD45^+^CD3^−^ population within CD45^+^/SS gated cells to exclude T cells and endothelial cells from analysis. Subsequently, cell subsets were first divided based on the expression of CD34 and CD117. From there, in a second step, each subset was analyzed for CD56 and CD94 expression. Using this gating strategy, we were able to identify seven distinctive developmental stages in BM (Fig. 1).

On this basis and in concert with NK cell developmental stages as identified in SLT [10], we now propose the following model of NK development (Table 2), starting from multi-potent CD34^+^CD117^−^CD56^−^CD94^−^ cells (stage 1), followed by the gain of CD117 (stage 2; CD34^+^CD117^+^CD56^−^CD94^−^). Subsequently, CD34 expression is lost in stage 3a (CD34^−^CD117^+^CD56^−^CD94^−^) followed by loss of multi-potency and acquirement of NK cell lineage commitment through CD56 acquisition in stage 3b (CD34^−^CD117^+^CD56^+^CD94^−^). After NK cell lineage commitment, cells gain CD94 expression and develop into immature CD56^bright^ NK cells (stage 4; CD34^−^CD117^+^CD56^+^CD94^+^). Through loss of CD117 expression, CD56^dim^ cells start to develop (stage 5a; CD34^−^CD117^−^CD56^+^CD94^+^), followed by loss of CD94 expression in stage 5b (CD34^−^CD117^−^CD56^+^CD94^−^). It should be noted, however, that stage 1 and stage 2 cells still obtain multi-lineage potential and therefore not only contain NK cell precursors but can also give rise to other cell lineages (e.g. T cells, DCs) [10].

**Table 2:** Developmental stages of NK cells in BM.

	CD34	CD117	CD56	CD94	CD56: bright or dim
Stage 1	+	−	−	−	-
Stage 2	+	+	−	−	-
Stage 3a	−	+	−	−	-
Stage 3b	−	+	+	−	CD56^dim^
Stage 4	−	+	+	+	CD56^bright^
Stage 5a	−	−	+	+	CD56^bright<dim^
Stage 5b	−	−	+	−	CD56^bright<<dim^

Main stages of NK cell development in BM based on expression profiles of CD34, CD117, CD56 and CD94 using flow cytometry panel 1 (Table 1).

### Early and sustained CD244 expression following in vivo NK cell developmental stages

**Figure 2:**
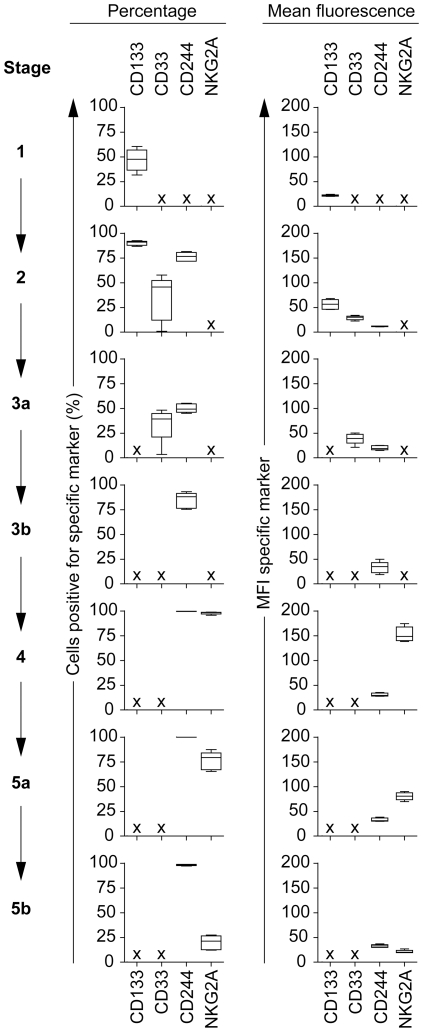
Expression of CD133, CD33, CD244 and NKG2A within the NK cell developmental stages in bone marrow (BM) (n = 5). Left panels show the percentages of cells positive for the specific markers. Right panels show the mean fluorescence (MFI) of each specific marker.

By using 10-color FCM, we were able to further specify the identified NK cell developmental stages in BM by analyzing additional antigen expression. For this purpose, we analyzed the cell surface expression of CD133, CD33, CD244 and NKG2A within each defined stage (Fig. 2). CD133 is known as a stem cell antigen which is expressed earlier in maturation than CD34 and, as such, may provide an alternative to CD34 for the selection and expansion of hematopoietic cells for transplantation [15]. Together with CD34, this antigen was only expressed within stages 1 and 2. CD33 has been described as an antigen for early NK cell development when showing lower fluorescence intensity as compared with its expression on myeloid cells [16] and was expressed in stages 2 and 3a. The CD244 receptor is suggested to be a co-receptor in activation of mature NK cells [17]. Interestingly, we found that CD244 was already expressed on CD34^+^CD117^+^ stage 2 cells in BM. During stages 3a and 3b, CD244 expression remained present and the amount of CD244^+^ cells was increased to more than 98% in stages 4 to 5b. Until now, CD244 expression was only shown to be present at early stages of human NK cell differentiation during *ex vivo* induced human NK cell maturation [18]. Recently, however, Fathman et al. showed that the expression of CD244 is linked to NK cell lineage commitment in an *in vivo* mouse model [19]. Thus, this suggests that NK cell lineage commitment may already be induced in part of early stage 2 and 3 cells, and that CD244 could be an important marker to dissect NK lineage committed cells from other lineage committed cells. The inhibitory NKG2A receptor, shown to be expressed early during NK cell maturation [13], was detected starting from stage 4 just after NK cell commitment (stage 3b) till stage 5b. In summary, as the different assessed antigens showed distinct expression profiles within the different stages, we were able to further refine the developmental stages as shown in Table 2 (Table 3), in which CD133 expression is expressed within stages 1 and 2, followed by CD33 expression in stages 2 and 3. In stage 2 and 3a, CD244 is upregulated followed by a continuous expression from stage 3b to 5b, and NKG2A is found in stages 4 to 5b on part of the cells.

**Table 3:** Developmental stages of NK cells in BM (continued).

	CD133	CD34	CD33	CD117	CD244	CD56	CD94	NKG2A	CD56: bright or dim
Stage 1	+/−	**+**	−	**−**	−	**−**	**−**	−	-
Stage 2	+	**+**	+/−	**+**	+/−	**−**	**−**	−	-
Stage 3a	−	**−**	+/−	**+**	+/−	**−**	**−**	−	-
Stage 3b	−	**−**	−	**+**	+	**+**	**−**	−	CD56^dim^
Stage 4	−	**−**	−	**+**	+	**+**	**+**	+	CD56^bright^
Stage 5a	−	**−**	−	**−**	+	**+**	**+**	+/−	CD56^bright<dim^
Stage 5b	−	**−**	−	**−**	+	**+**	**−**	+/−	CD56^bright<<dim^

Further identification of developmental NK cell stages in BM based on expression of CD133, CD34, CD33, CD177, CD244, NKG2A, CD56 and CD94 using flow cytometry panel 1 (Table 1). Indicated is the presence of each specified marker within each stage (based on the percentage of positive cells present): + = 100–80%; +/−<80%; − = below reliable detection limits.

### NK cell development starts in BM, followed by further maturation in LN, SPL and PB

**Figure 3:**
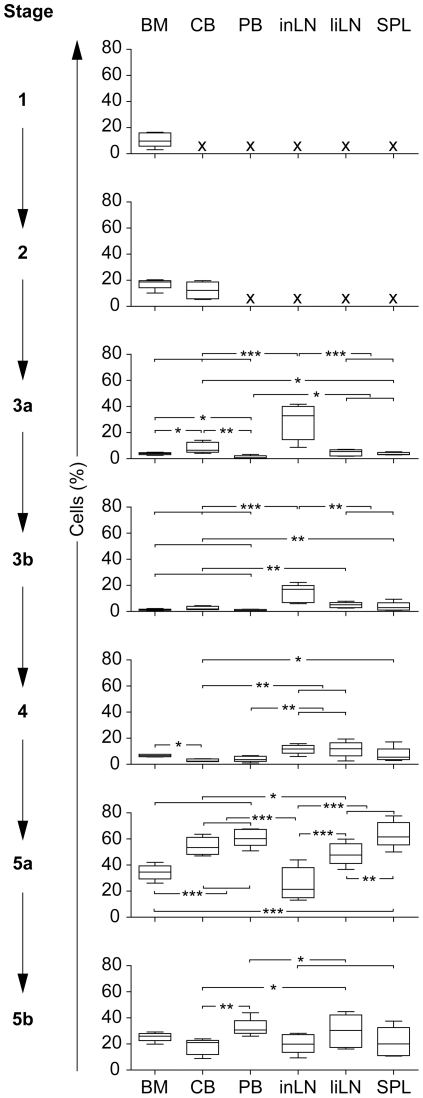
Distribution of the NK cell developmental stages in different human tissues. Shown are the results for bone marrow (BM), cord blood (CB), peripheral blood (PB), inguinal LN (inLN), liver LN (liLN) and spleen (SPL) (all n = 5). Shown are all NK cell developmental stages within each tissue; *P<.05, **P<.01, ***P<.0001.

To assess whether the NK developmental stages can be found in other human tissues besides BM, we further analyzed samples of cord blood (CB), peripheral blood (PB), inguinal LN (inLN), liver LN (liLN) and spleen (SPL) (Fig. 3). Results showed a differential distribution of the NK cell developmental stages within the different tissues. The NK cell developmental stages in BM mainly consisted of stage 5a and 5b cells. In addition, stages 1 and 2 were only detected in BM, confirming BM as the origin of NK cell development. In CB, stage 2 cells were found, but not in PB, suggesting that blood of fetal origin may possibly contain more early NK progenitor cells as compared with adult blood. However, the main NK cell developmental stages in CB and PB were stage 5a and 5b cells. In contrast to other tissues, the distribution of NK cell developmental stages in inLN primarily contained stage 3a and stage 3b cells, and showed lower, but similar, frequencies of stages 4 to 5b. In contrast, NK cell developmental stages in liLN and SPL consisted primarily of stages 4, 5a and 5b cells.

To exclude that stage 3a and 3b consisted largely, not of potential precursor NK cells, but rather of lymphoid tissue inducer cells (LTIs; RORγt^+^NKp44^+^CD127^+^) [20], we additionally stained two inLN and liLN samples for stage 3 markers combined with RORγt, NKp44 and CD127 mAbs. Results showed that stage 3a in inLN and liLN contained only 0.8–15% and 3.9–4.8% LTIs, respectively. Stage 3b in inLN and liLN contained 7–21% and 13–19% LTIs, respectively (data not shown). This suggests that stage 3a and 3b in the lymphoid compartments may primarily consist of precursor NK cells.

Following the presence of the different NK developmental stages within the different tissues analyzed, our results suggest that early NK progenitor cells may potentially migrate from BM to SLT, after which pre-NK cells (stage 3a) may further develop in LN leadings to NK cell commitment (stage 3b), followed by further maturation in splenic tissue and the release of mature NK cells into the blood stream. The presence of different stages within one tissue, for instance stages other than stage 1 and 2 in BM or stage 3 in LN, suggests that *in situ* differentiation of remaining cells may also occur besides potential trafficking of developmental stages towards other tissues.

### Sustained CD33 expression in liLN following in vivo NK cell developmental stages

**Figure 4:**
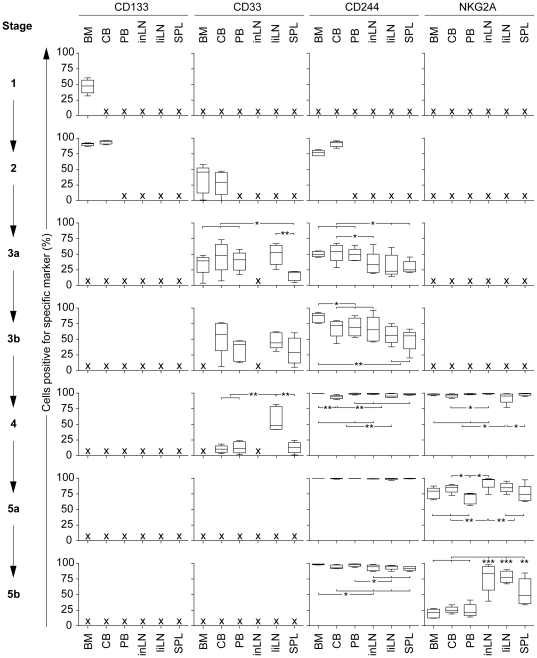
Expression of CD133, CD33, CD244 and NKG2A within the NK cell developmental stages. Shown are the results for bone marrow (BM), cord blood (CB), peripheral blood (PB), inguinal LN (inLN), liver LN (liLN) and spleen (SPL) (all n = 5); *P<.05, **P<.01, ***P<.0001.

To assess potential differences of the NK cell developmental stages within the human tissues, we further analyzed the expression of CD133, CD33, CD244 and NKG2A within the stages present in the human tissues (Fig. 4). Between BM and CB, there were no significant differences in expression of CD133, CD33 and CD244 within stage 2. The subsequent trend of CD244 acquisition was comparable for each tissue and all tissues showed more than 98% CD244^+^ cells in stages 4 and 5a/b. Significant differences were seen in the expression profile of the early CD33 antigen within the different human tissues. As we previously characterized CD33 expression to be specific for stage 2 and 3a cells in BM (Table 3), CD33 expression was prolonged in CB, PB, and SPL until stage 3b. Furthermore, in liLN, CD33 expression was even sustained after NK cell commitment until stage 4. The prolonged expression of CD33 in some distinct stages and tissues may be due to tissue specific NK cell developmental subsets *in situ*.

### NKG2A expression reveals an impaired NK cell maturation profile in lymphoid tissues

Following the evaluation of the tissue specific NK cell subsets, by the expression profile of CD33, we subsequently studied whether the tissue specific differences are also expressed in the NK cell maturation pattern. As the level of NKG2A expression may be representative for the level of NK cell maturation [21], [22], we analyzed the NKG2A expression profile on “committed” NK cells. Besides the significant differences in the CD33 expression profile, the expression profile of NKG2A also showed a distinction between the different human tissues. In stage 4, all tissues contained more than 95% NKG2A^+^ cells. Following NK cell developmental stages, BM, CB and PB showed a decrease in the percentage of NKG2A^+^ cells up to approximately 25% in stage 5b, whereas in both LN a median of 75–80% remained NKG2A^+^ and SPL kept a median of 50% NKG2A^+^ cells. The stronger decrease of NKG2A^+^ cells in BM, CB and PB as compared with other tissues was also reflected in the mean fluorescence intensity (MFI) of NKG2A expression following stage 4 to 5b (Fig. S1). Overall, these data suggest that the committed NK cells in LN and SPL have a more immature phenotype as compared with cells present in BM, PB and CB.

In order to better define NK cell maturation, we extended our analyses with regard to “committed” NK cells. Therefore, we subsequently analyzed the expression of additional NK cell receptors to further asses the maturity status of the committed NK cells within the different human tissues.

### Differences in the NK cell receptor repertoire suggest distinct in situ NK cell development within LN and CB

Phenotypically committed NK cells (CD45^+^CD3^−^CD56^+^) can generally be divided into two distinguishable subsets: the CD56^bright^CD16^+/−^ and the CD56^dim^CD16^+^ subset [23]. Our data confirmed the heterogeneity of the CD56^bright^CD16^+/−^ and CD56^dim^CD16^+^ subsets within BM, CB, PB, and LN, showing balances of CD56^bright^>>CD56^dim^ in LN, and CD56^bright^<<CD56^dim^ in BM, CB and PB (Fig. S2). Additionally, we identified a CD56^bright^≈CD56^dim^ balance in SPL.

To further assess the maturity of the committed NK cell subsets, we analyzed the expression of killer immunoglobulin-like receptors (KIR), NKG2A/C, NKG2D, CD244 and natural cytotoxicity receptors (NCR; NKp30, NKp44, NKp46) within the CD45^+^CD56^+^CD3^−^ population by using FCM panels 2 and 3 (Table 1). These receptors trigger and modulate mature NK cell effector function through a balance between inhibitory (KIR, NKG2A) and stimulatory signals (NKG2C, NKG2D, CD244, NCR) [2], [24].

**Figure 5:**
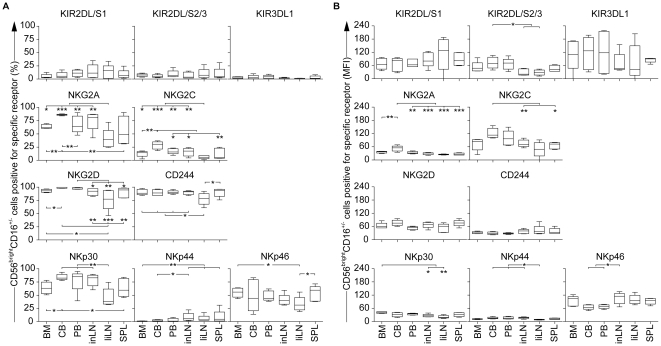
Expression of KIR, NKG2A/C, NCR, NKG2D and CD244 within the CD56^bright^CD16^+/−^ NK cell subset of committed NK cells in bone marrow (BM), cord blood (CB), peripheral blood (PB), inguinal LN (inLN), liver LN (liLN) and spleen (SPL) (all n = 5). (A) Shown are the percentages of CD56^bright^CD16^+/−^ cells positive for each specific receptor within each tissue; *P<.05, **P<.01, ***P<.0001. (B) Shown is the mean fluorescence intensity (MFI) for each specific receptor expressed by CD56^bright^CD16^+/−^ cells; *P<.05, **P<.01, ***P<.0001.

**Figure 6:**
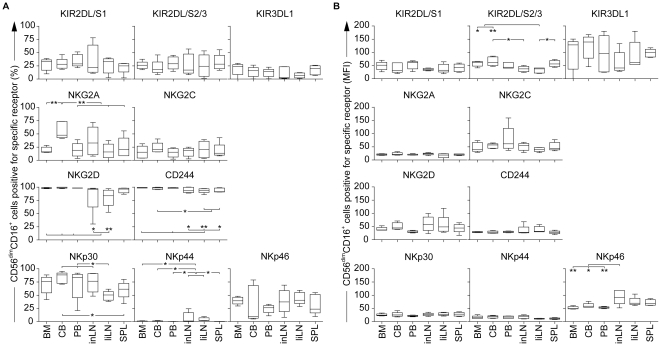
Expression of KIR, NKG2A/C, NCR, NKG2D and CD244 within the CD56^dim^CD16^+^ NK cell subset of committed NK cells in bone marrow (BM), cord blood (CB), peripheral blood (PB), inguinal LN (inLN), liver LN (liLN) and spleen (SPL) (all n = 5). (A) Shown are the percentages of CD56^dim^CD16^+^ cells positive for each specific receptor within each tissue; *P<.05, **P<.01. (B) Shown is the mean fluorescence intensity (MFI) for each specific receptor expressed by CD56^dim^CD16^+^ cells; *P<.05, **P<.01.

We first analyzed the NK cell receptor repertoire of the CD56^bright^CD16^+/−^ subset within the committed NK cell population of each tissue (Fig. 5). Results showed that there was no difference in the amount of KIR^+^ cells between the tissues. Nevertheless, the MFI of KIR2DL/S2/3 and KIR3DL1 was lower in both LN and SPL, suggesting a more immature phenotype of CD56^bright^ cells as compared with BM, CB and PB. Surprisingly, the proportion of NKG2A^+^ cells was significantly lower in liLN as compared to other tissues. This may be explained by a different NK cell development *in situ*, as suggested by the prolonged expression of CD33 (Fig. 4). Furthermore, the amount of activating receptor positive cells, with the exception of NKp44, was also lower in liLN as compared with other tissues. This was also reflected within the CD56^dim^CD16^+^ subset of liLN, showing lower amounts of NKG2D^+^, CD244^+^ and NKp30^+^ cells as compared with other tissues (Fig. 6). Thus, these results suggest that NK cell development in LN may differ *in situ* between LN at different anatomical locations and also other tissues.

Analysis of CB showed that both the CD56^bright^CD16^+/−^ (Fig. 5) and the CD56^dim^CD16^+^ (Fig. 6) subset contained significantly more NKG2A^+^ cells as compared with other tissues. In addition, the level of NKG2A expression (MFI) in the CD56^bright^CD16^+/−^ subset was also significantly higher, which confirmed previous results [25]. NKG2C, which is the stimulatory lectin-like counterpart of NKG2A, also showed elevated expression within the CD56^bright^CD16^+/−^ subset of committed NK cells in CB (Fig. 5). These data suggest that the fetal micro-environment of CB may provide prevalence for the expression of lectin-like antigens as compared with other human tissues.

Overall, the data on the NK cell receptor repertoire within the different subsets of the committed NK cells demonstrates the heterogeneity of the CD56^bright^CD16^+/−^ and CD56^dim^CD16^+^ within the different compartments and suggests that microenvironment may play a role in differential *in situ* development of the NK cell receptor repertoire of committed NK cells.

## Discussion

In contrast to T and B cells, the developmental pathways and locations for human NK cell development are currently less well defined. Results of this study suggest that this is partly due to the heterogeneity of NK cell subsets and their diverse anatomical distribution over the different compartments within the human body. Using 10-color FCM, we were able to distinguish seven NK cell developmental stages, which are numbered in line with the initial 4 stages as described by Freud *et al*. [10] and that we further specified by the expression profiles of CD133, CD33, CD244 and NKG2A. It should be noted, however, that of the seven stages that we detected, stages 1 and 2 still obtain multi-lineage potential, whereas NK cell lineage commitment is described to be completed in stage 3 [10]. Our results showed that NK cell development may be accompanied by early expression of CD244 starting at stage 2–3 and that there is a diverse distribution of the NK cell developmental stages among different human tissues. In addition, we provided a detailed phenotypical characterization of the committed NK cell population revealing different NK cell maturation stages (i.e. CD56^bright^:CD56^dim^ ratio and heterogeneity in the NK cell receptor repertoire) within the tissues.

Our data pointed that NK cell development originates from BM as stage 1 and 2 cells (CD34^+^CD117^−^CD56^−^CD94^−^ and CD34^+^CD117^+^CD56^−^CD94^−^, respectively) were only found in BM and not in LN, SPL or PB. This is in contrast to the findings of by Freud *et al.*, who identified stage 1 and 2 cells in SLT [10]. This may be due to differences in sample preparation before analysis. In the previous study, for each donor tissue, cells from multiple tonsil pieces or multiple LN were pooled in order to obtain sufficient numbers within each NK cell developmental stage. In contrast, with the use of 10-color flow cytometry, we were able to study small sample sizes of tissues obtained from single donors. Due to biological variation between donors, pooled samples of each tissue may cause a non-representative view on NK cell subset numbers present within the different tissues analyzed. However, due to the detection limits that we set for our analysis, we cannot rule out that very small populations remained undetected within our samples. Nevertheless, without pooling samples, we believe that this unique collection of tissue samples provides a genuine view on the distribution of the different NK cell developmental stages within the different anatomical locations as they are *in vivo*.

Upon acquiring CD56 expression (NK cell commitment), the cells acquired CD94 expression. Simultaneously, NKG2A positive cells appeared. Later, following NK cell development, CD94 expression decreased during the final maturation steps towards a CD56^dim^ phenotype together with a decrease in NKG2A expression [16], [26]. This phenomenon has also been described in the setting of allogeneic SCT. Following NK cell repopulation after allogeneic SCT, we and others have shown that CD56^bright^ cells are the first committed NK cells to arise in the early phase after transplantation followed by the repopulation of CD56^dim^ cells suggesting a CD56^bright^ to CD56^dim^ developmental pathway [13], [14]. This has been further supported by research of Dulphy *et al.* in which an intermediate CD56^bright^CD16^low^ population was found in the early phase after allogeneic SCT before repopulation of the CD56^dim^CD16^+^ subset [27]. Overall, our findings described in this paper are in line with the *in vivo* repopulation of NK cells after allogeneic SCT and therefore support a model in which CD56^bright^ cells further develop into CD56^dim^ cells which may ultimately result in the CD56^bright^<<CD56^dim^ ratio as seen in the periphery of healthy individuals. The concomitant downregulation of NKG2A with CD94 during the final maturation steps towards a CD56^dim^ phenotype may be explained by the fact that these two molecules form a functional inhibitory NKG2A/CD94 heterodimer recognizing the ubiquitously expressed HLA-E molecule [28], [29]. Notably, we also observed a mature *in vivo* NK cell developmental stage, that primarily consisted of CD56^dim^ cells, expressing low levels of NKG2A without CD94 (stage 5b). The low expression level of NKG2A in combination with the fact that NKG2A only contributes to the binding affinity of the heterodimeric complex and HLA-E solely interacts with CD94 [29], suggests that in this case NKG2A is non-functional. Recently, the functional NKG2A/CD94 receptor complex was described to be endocytosed by a macropinocytic-like process, which may be related to the maintenance of its surface expression [30]. However, as it is not clear whether the NKG2A/CD94 complex is internalized as a whole or may be first uncoupled before internalization, it may be possible that for a brief period NKG2A may exist on the cell surface without CD94. Together, this suggests that inhibition of cells in stage 5b largely depends on KIR signaling, and that stage 5b is one of the final stages in NK cell development.

Remarkably, the phenotype of committed NK cells (stage 3b-5b) differed between both LN (liver and inguinal sites). The NK cell receptor repertoire in liLN showed significantly less cells expressing NKG2A/C, NKp30, CD244 and NKG2D. All together, these results suggest that committed NK cells in liLN reside in an even more immature state as compared with committed NK cells in inLN. This was confirmed by the expression of CD33, a marker for early NK cell development, which is expressed significantly longer by committed NK cells in liLN as compared with inLN and other tissues following NK cell development. Similarly, CD56^bright^ NK cells in the liver itself show a more immature phenotype as compared to their counterpart in PB [31]. The differences in NK cell differentiation *in situ* after NK cell commitment may be due to the regional immune system of the liver, which is characterized by relatively weak cellular immune responses and hyporesponsiveness to antigens and bacterial product derived from the intestines [32]. As NK cells form a bridge between innate and adaptive immune responses, the immature state of committed NK cells in liver and its draining LN may play a role in this. However, as we were not able to perform functional analysis due to small sample sizes, this remains subjected to further study.

Our findings confirm results from previous studies showing that the CD56^bright^CD16^+/−^ and CD56^dim^CD16^+^ subsets are present in the same proportions in both CB and PB [33], [34]. Nevertheless, these studies also reported that CB NK cells have a natural reduced killing ability as compared with PB NK cells. We postulate that this may be due to differences in NKG2A expression as our results showed that NKG2A is significantly more expressed in CB as compared with PB and also other tissues. Nevertheless, the reduced killing ability of CB NK cells can be reversed after cytokine stimulation [33], [34]. Thus, as CB is a source for *ex vivo* generation of NK cell-based immunotherapeutics [35], the high expression of NKG2A and its inhibitory effect on CB NK cell function may not necessarily form a problem for NK cell-based immunotherapeutic strategies.

Although our analysis of the committed NK cells could not distinguish NK cell subsets between stages 3b to 5b, the ratio between the CD56^bright^CD16^+/−^ and CD56^dim^CD16^+^ subsets and the overall expression of KIR and NKG2A provided a clear overview on the maturation status of committed NK cells within the different tissues. Committed NK cells in LN represented the most immature status as the NK cells profoundly consisted of CD56^bright^CD16^+/−^ cells, followed by SPL showing an equal balance of both subsets. BM, CB and PB held the most mature status of NK cells as they abundantly contained the CD56^dim^CD16^+^ subset. After allogeneic SCT, *in vivo* maturation of the NK cell receptor repertoire is characterized by fast upregulation of NKG2A followed by the acquisition of KIR together with a slow decrease in NKG2A expression [13], [14], [21], [36]. The maturation status of the committed NK cells within the different tissues analyzed confirm these findings as the overall KIR expression was highest in BM, CB and PB, followed by SPL and was lowest in LN. With the exception of CB, ranges of NKG2A expression were lowest in BM and PB, followed by a higher range of NKG2A expression in LN and SPL.

**Figure 7:**
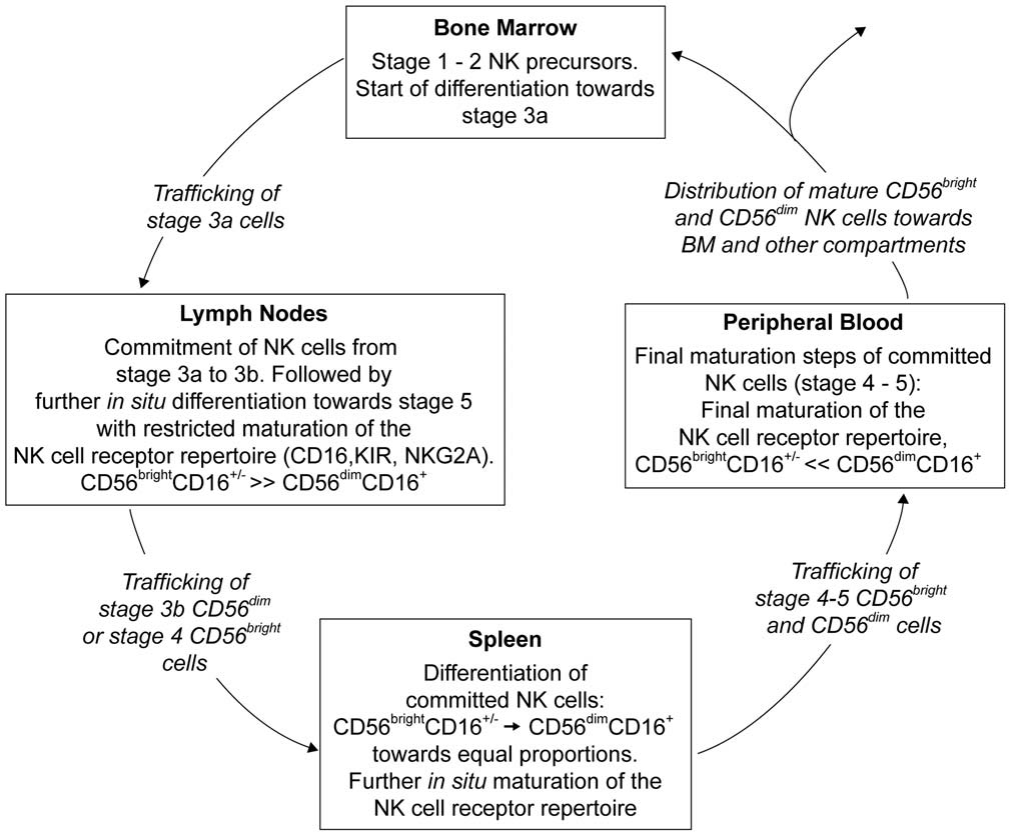
Proposed model for human NK cell development *in vivo*. Based on our data, we propose that precursor NK cells (stage 2) may traffic from BM to LN, where commitment to the NK cell lineage may take place (stage 3a→3b) followed by potential *in situ* differentiation of NK cells with restricted maturation of the NK cell receptor repertoire. For further differentiation of committed NK cells, CD56^dim^ (stage 3b) or CD56^bright^ cells (stage 4) may traffic towards splenic tissue in which CD56^dim^ cells may develop and further maturation of the NK cell receptor repertoire can take place. Final maturation of NK cells may occur through trafficking of cells towards the periphery from which NK cells may be further distributed to different compartments in the human body.

In summary, these data may support a model for *in vivo* NK cell development indicating BM as the origin of NK cell development (Fig. 7). Through trafficking of precursor NK cells from BM to LN, commitment to the NK cell lineage may take place in LN followed by potential *in situ* differentiation and restricted maturation of the NK cell receptor repertoire. For further differentiation of committed NK cells, stage 3b CD56^dim^ cells or stage 4 CD56^bright^ cells may traffic towards splenic tissue in which stage 5 cells could develop and further maturation of the NK cell receptor repertoire can take place. Final maturation of NK cells may occur through trafficking of cells (stage 4 to 5) towards the periphery from which NK cells may be further distributed to different compartments in the human body. Although the phenotypical data presented in this paper are of course no direct evidence for the proposed model, this is the first study in which the presence of different NK cell developmental stages and their mature subsets are analyzed within a unique set of healthy human tissue samples in great detail. Therefore, our results may serve as a valuable and important basis for future NK cell developmental studies as well as newly developed NK cell generation protocols that are being transferred towards clinical use. Nevertheless, further functional analyses of developing and committed NK cells within the different NK cell developmental stages are clearly warranted to support our findings and to obtain a complete view on the NK cell developmental pathway that includes the acquisition of the cytolytic and cytokine producing functions during NK cell development.

## Materials and Methods

### Tissue collection and mononuclear cell isolation

Bone marrow (BM), peripheral blood (PB), spleen (SPL) and cord blood (CB) samples were obtained at the Radboud University Nijmegen Medical Centre (RUNMC; Nijmegen, The Netherlands). BM and PB samples were obtained from healthy SCT donors before mobilization treatment with G-CSF. SPL samples were obtained from deceased liver or kidney transplantation donors. CB samples, obtained at birth after normal full-term delivery, were provided by the cord blood bank of RUNMC. At the Erasmus Medical Centre (Rotterdam, The Netherlands), liver draining lymph node (LiLN) samples were obtained from deceased liver transplantation donors and inguinal lymph node (inLN) samples from kidney transplant recipients (not treated with immunosuppressive drugs prior to LN excision). After collection, each tissue sample was stored at room temperature and processed within 24 h. LN and SPL samples were first forced through 74 µm netwell filters (Costar, Corning International, NY, and USA) to obtain single cell suspensions. Mononuclear cells (MNC) were isolated by density gradient centrifugation (Lymphoprep; Nycomed Pharma, Roskilde, Denmark) and cryopreserved in liquid nitrogen until further use. At least 5 independent samples of each tissue were collected. This study was performed in accordance with the regulations set by the Medical Ethical Committees for human research of the RUNMC and the Erasmus MC. The Medical Ethical Committees for human research of the RUNMC and the Erasmus MC have approved the use of this material for the current study.

Written informed consent with regard to scientific use was obtained from all study participants or their representatives.

### Multi-color flow cytometry

For analysis, we designed three different 10-color FCM panels (Table 1) using conjugated mAbs kindly provided by Beckman Coulter (Marseille, France) with the exception of CD16-FITC (Dako, Glostrup, Denmark) and CD159c-PE (R&D Systems, Minneapolis, CA, USA). Combinations of mAb-fluorochromes were balanced to avoid antibody interactions, sterical hindrance and to detect also dimly expressing populations. Before multi-color analyses, all conjugates were titrated and individually tested for sensitivity, resolution and compensation of spectral overlap. Isotype controls were used to define marker settings. Thawed MNC fractions of collected human tissues were assessed on a Navios™ flow cytometer and analyzed using Kaluza Software® 1.0 (Beckman Coulter). To define NK cell developmental stages, samples were gated on the CD45^+^CD3^−^ population within CD45^+^/SS gated cells to exclude T cells and endothelial cells (which may express CD34 but are CD45 negative [37]) and debris from analysis. To analyze the NK cell receptor repertoire of committed NK cells, cells were further gated on CD56^+^ cells within the CD45^+^CD3^−^ population. An additional staining was performed to check for the presence of lymphoid tissue inducer cells (LTIs) in the lymphoid tissues [20]. To this end, a surface staining was performed using CD34-FITC, CD336-PE, CD3-ECD, CD127-PE-Cy5, CD117-PC7, CD56-APC-A750, CD94-PB and CD45-PO conjugated mAbs followed by an intracellular staining of RORγt using an APC-conjugated mAb (clone AFKJS-9; eBioscience, San Diego, CA, USA) together with Fix and Fix/Perm buffer (eBioscience, San Diego, CA, USA) according to the manufacturer's instructions. LTIs were identified within the CD45^+^CD3^−^ population as CD34^−^CD117^+^CD94^−^CD56^+/−^ cells expressing CD127, NKp44 and RORγt.

Cell populations >0.1% of the CD45^+^CD3^−^ population with a threshold of more than 50 cells were considered reliable. Cell populations were considered to be present in a specific tissue when at least 3 out of 5 samples showed reliable results. Cell populations that did not suffice to these criteria were excluded from further (statistical) analysis. An overview of analyzed sample sizes is shown in Table S1.

### Statistical analysis

To compare percentages of cells positive for single markers between the different tissues, a random effect logistic regression model was used that accounted for the biological diversity between samples of each tissue and for the fact that several samples of each tissue type were taken. Mean fluorescence (MFI) of specific markers between the different tissues were analyzed using ANOVA analysis with Tukey post testing. *P*-values <0.05 were considered significant.

## Supporting Information

Figure S1 NKG2A expression levels in stages 4, 5a, and 5b in bone marrow (BM), cord blood (CB), peripheral blood (PB), inguinal LN (inLN), liver LN (liLN) and spleen (SPL) (all n = 5); * ***P*** <.05, ** ***P*** <.01, *** ***P*** **<.0001.**
(TIF)

Figure S2 **CD56 and CD16 expression patterns of committed NK cells (stage 3b-5b) within different human tissues.** BM = bone marrow, CB = cord blood, PB = peripheral blood, inLN-inguinal LN, liLN = liver LN, SPL = spleen (all n = 5). (A) Shown are representative examples (one of each tissue) for CD56 and CD16 expression patterns within the committed NK cell population. (B) Shown are the distribution of the CD56^bright^CD16^+/−^ subset (left panel) and the CD56^dim^CD16^+^ subset within the different human tissues; *P<.05, **P<.01, ***P<.0001.
(TIF)

Table S1 **To define NK cell developmental stages, samples were gated on the CD45^+^CD3^−^ population within CD45^+^/SS gated cells to exclude T cells and endothelial cells from analysis.** For each tissue, the following items are indicated: ^1^Total cell number within the CD45^+^/SS gate; ^2^the amount of cells within the CD45^+^CD3^−^ gate and; ^3^the total amount of cells covering all NK cell developmental stages. All cell numbers are shown in median (range).
(DOC)
