# Supplementary figures and images for: Defining Early Human NK Cell Developmental Stages in Primary and Secondary Lymphoid Tissues
Source: PLoS One. 2012 Feb 3;7(2):e30930. doi: 10.1371/journal.pone.0030930 (PMC3272048; doi:10.1371/journal.pone.0030930)

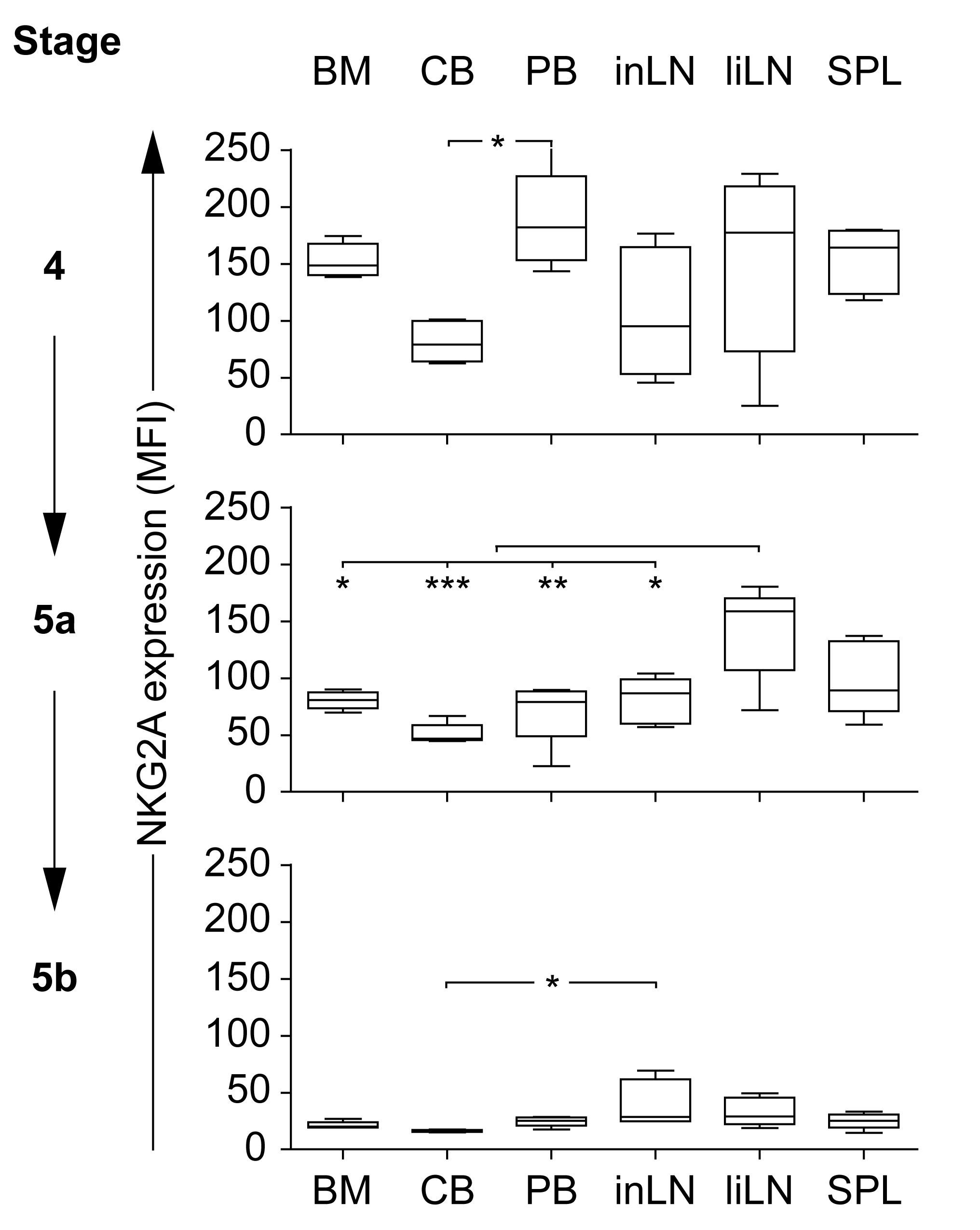

Supplement: Figure S1 — NKG2A expression levels in stages 4, 5a, and 5b in bone marrow (BM), cord blood (CB), peripheral blood (PB), inguinal LN (inLN), liver LN (liLN) and spleen (SPL) (all n = 5); * P <.05, ** P <.01, *** P <.0001. (TIF) [file pone.0030930.s001.tif]

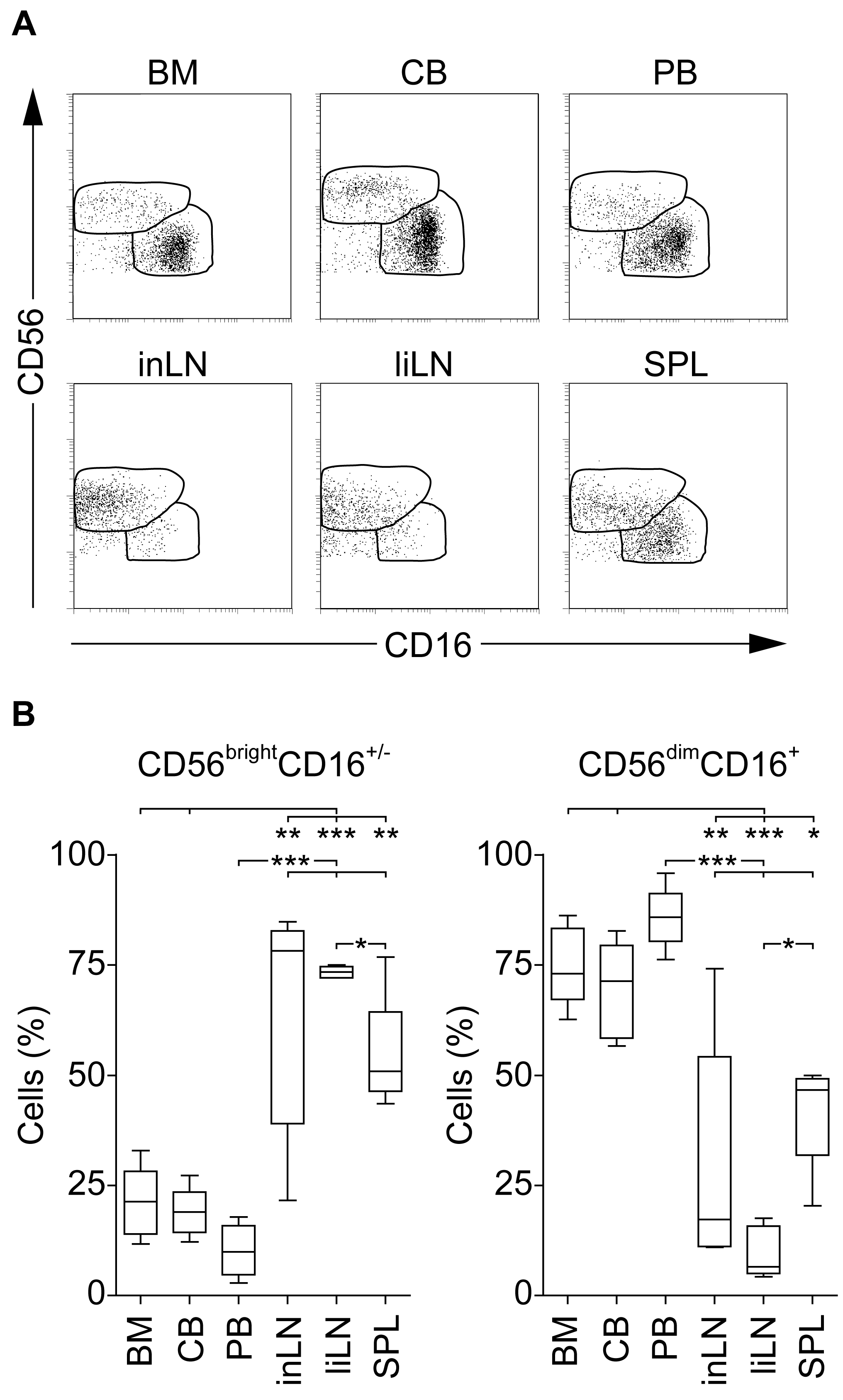

Supplement: Figure S2 — CD56 and CD16 expression patterns of committed NK cells (stage 3b-5b) within different human tissues. BM = bone marrow, CB = cord blood, PB = peripheral blood, inLN-inguinal LN, liLN = liver LN, SPL = spleen (all n = 5). (A) Shown are representative examples (one of each tissue) for CD56 and CD16 expression patterns within the committed NK cell population. (B) Shown are the distribution of the CD56brightCD16+/− subset (left panel) and the CD56dimCD16+ subset within the different human tissues; *P<.05, **P<.01, ***P<.0001. (TIF) [file pone.0030930.s002.tif]
