# Supplementary material for: Defining Early Human NK Cell Developmental Stages in Primary and Secondary Lymphoid Tissues
Source: PLoS One. 2012 Feb 3;7(2):e30930. doi: 10.1371/journal.pone.0030930 (PMC3272048; doi:10.1371/journal.pone.0030930)
Supplement: Table S1 — To define NK cell developmental stages, samples were gated on the CD45+CD3− population within CD45+/SS gated cells to exclude T cells and endothelial cells from analysis. For each tissue, the following items are indicated: 1Total cell number within the CD45+/SS gate; 2the amount of cells within the CD45+CD3− gate and; 3the total amount of cells covering all NK cell developmental stages. All cell numbers are shown in median (range). (DOC) [file pone.0030930.s003.doc]

|  | Total cell number^1^ (x10^4^) | Gated CD45^+^CD3^-^ cells^2^ (x10^4^) | Progenitor cells^3^ (x10^4^) |
| --- | --- | --- | --- |
| BM | 16.9 (8.9-34.9) | 2.8 (1.9-3.3) | 0.98 (0.7-1.2) |
| CB | 39.9 (27.5-99.9) | 12.2 (7.1-31.4) | 1.88 (0.6-5.0) |
| PB | 37.8 (35.7-38.9) | 8.8 (5.4-11.0) | 4.15 (1.1-7.7) |
| inLN | 10.0 (21.3-61.9) | 6.3 (1.2- 34.4) | 2.2 (0.4-5.4) |
| liLN | 4.0 (2.5-39-6) | 7.8 (1.5-29.7) | 1.1 (0.1-3.0) |
| SPL | 40.8 (27.4-45.8) | 25.2 (13-29.8) | 3.03 (2.1-4.1) |
